# Impact of hepatocellular carcinoma heterogeneity on computed tomography as a prognostic indicator

**DOI:** 10.1038/s41598-017-12688-7

**Published:** 2017-10-04

**Authors:** Shigeru Kiryu, Hiroyuki Akai, Masanori Nojima, Kiyoshi Hasegawa, Hiroji Shinkawa, Norihiro Kokudo, Koichiro Yasaka, Kuni Ohtomo

**Affiliations:** 1 Department of Radiology, International University of Health and Welfare Hospital, Nasushiobara, Tochigi, Japan; 2 Department of Radiology, The Institute of Medical Science, The University of Tokyo, Tokyo, Japan; 3 Division of Advanced Medicine Promotion, The Advanced Clinical Research Center, The Institute of Medical Science, University of Tokyo, Tokyo, Japan; 4 Division of Hepato-Biliary-Pancreatic Surgery, Department of Surgery, Graduate School of Medicine, University of Tokyo, Tokyo, Japan; 5 International University of Health and Welfare, Otawara, Tochigi, Japan

## Abstract

We assessed the relationship between the heterogeneity of HCC on preoperative non-contrast-enhanced CT and patient prognosis. The heterogeneity of CT images from 122 patients was assessed and texture feature parameters such as mean, standard deviation (SD), entropy, mean of the positive pixels (MPP), skewness, and kurtosis were obtained using filtration. The relationship between CT texture features and 5-year overall survival (OS) or disease-free survival (DFS) was assessed. Multivariate regression analysis was performed to evaluate the independence of texture feature from clinical or pathological parameters. The Kaplan-Meier curves for OS or DFS was significantly different between patient groups dichotomized by cut-off values for all CT texture parameters with filtration at at least one filter level. Multivariate regression analysis showed the independence of most CT texture parameters on clinical and pathological parameters for OS with filtration at at least one filter level and without filtration except kurtosis. SD, entropy, and MPP with coarse filter, and skewness without filtration showed a significant correlation for DFS. CT texture features of non-contrast-enhanced CT images showed a relationship with HCC prognosis. Multivariate regression analysis showed the possibility of CT texture feature increase the prognostic prediction of HCC by clinical and pathological information.

## Introduction

The incidence of hepatocellular carcinoma (HCC) has increased due to the spread of hepatitis, and HCC is now the second leading cause of cancer-related death worldwide^1^. Because HCC has a high potential for vascular invasion, metastasis, and recurrence, its prognosis is poor. Among several therapies used to treat HCC, hepatic resection is considered the mainstay of curative therapy for localized HCC^2,3^. However, due to its high rate of recurrence after resection, preoperative prognostic prediction of HCC is important for the appropriate management of patients.

Heterogeneity is widely recognized as a feature of malignancy associated with adverse tumor biology, and it is assessed on medical images using a texture-analysis technique^4^. On computed tomography (CT), photon noise, caused by fluctuations in the number of photons in incident X-rays, obscures biologic heterogeneity^4^. CT texture analysis using filters to reduce photon noise is used for a variety of purposes, including assessment of malignant tumor prognosis^5^.

CT is widely used for the detection and assessment of HCC and is an indispensable preoperative examination. If information related to HCC prognosis is available from non-contrast-enhanced CT images, preoperative CT can provide additional meaningful data. The objective of this study was to assess the relationship between HCC heterogeneity and prognosis after the resection of HCC using non-contrast-enhanced CT texture analysis with a filter technique.

## Materials and Methods

This study was approved by the Research Ethics Committee of our institution as a retrospective data analysis for medical imaging-based diagnoses, and the requirement for informed consent was waived by the Committee (26-73-1126). Additionally, all experiments and methods in this study were performed in accordance with the Declaration of Helsinki.

### Patients

A total of 169 consecutive patients who underwent initial hepatectomy for HCC between January 2004 and September 2009 were included in this study. This study was approved by the Research Ethics Committee of the institution. A total of 16 patients were excluded due to the unavailability of CT images on the picture archiving system. We excluded a further 28 patients who underwent transcatheter arterial embolization (TACE) prior to CT, because the high density of deposited iodized oil may affect the texture features. A total of three patients with lesions too small (<1 cm) to evaluate on CT were additionally excluded. Finally, the study group consisted of 122 patients: 92 males (mean age, 63.5 years; range, 25–82 years) and 30 females (mean age, 69.2 years; range, 53–81 years). A total of 37 patients underwent TACE between CT and surgery (Table 1). Clinical patient’s information such as Child-Pugh score, serum levels of alpha-fetoprotein (AFP), tumor differentiation, pathological stage (pStage), or presence/absence of venous invasion was examined using database of surgical records.

**Table 1:** Patient’s background.

Gender (male/female)	92 (75.4%)/30 (24.5%)
Age (years)	69.2
HBs-Ag	27 (22.1%)
HCV-Ab	60 (49.2%)
Cirrhosis	59 (48.4%)
Child-Pugh A/B	106 (86.9%)/16 (13.1%)
AFP (ng/ml) >400/< = 400	22 (18.0%)/100 (82.0%)
Differentiation	
well/moderate/poor/necrosis	26 (21.3%)/82 (67.2%) 10 (8.2%)/4 (3.3%)
TNM stage	
I/II/III/IVA/IVB	12 (9.8%)/60 (49.2%)/35 (28.7%)/12 (9.8%)/3 (2.5%)
Tumor size (cm)	7.1
Tumor number 1/2/>3	77 (63.1%)/24 (19.7%)/21 (17.2%)
Venous invasion	38 (31.1%)
Preoperative TACE cases	37 (30.3%)

Values are presented as number,or number (%).

### CT examination

CT studies were performed using a 4-, 16-, or 64-detector row CT scanner (Light Speed QX/I, LightSpeed Ultra, LightSpeed VCT, GE Medical Systems, Milwaukee, WI, USA) or a 16-detector row CT scanner (Aquilion 16, Toshiba Medical Systems Corp., Tochigi, Japan). The same clinical protocol was used: 120 kV, 180–280 mAs depending on body habitus, a matrix of 512, and a field of view of 350–400 mm. Non-contrast CT was performed using a 5-mm contiguous axial section to encompass the whole liver. Non-contrast CT was mostly performed as part of the contrast-enhanced study.

### Texture analysis

**Figure 1:**
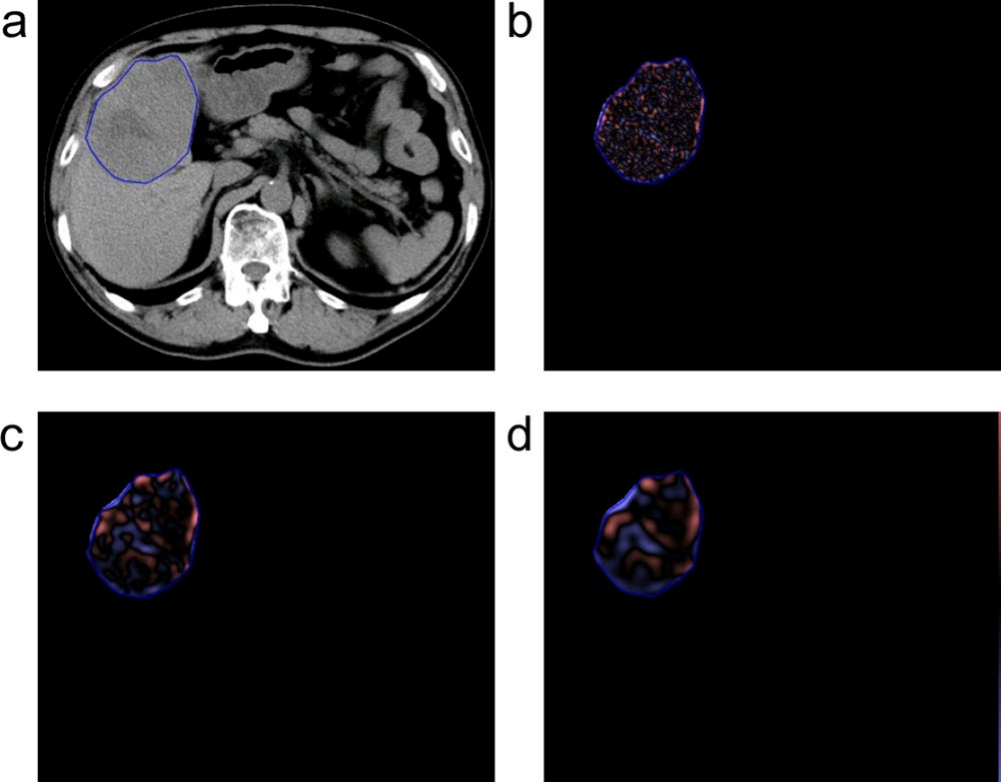
Texture analysis of computed tomography (CT) images of hepatocellular carcinoma (HCC). (**a**) Preoperative non-contrast-enhanced CT image obtained before hepatic resection shows a region of interest drawn around the periphery of the HCC. A corresponding post-processed CT image displays (**b**) fine, (**c**) medium, and (**d**) coarse textures obtained at fine (2 mm wide), medium (4 mm wide), and coarse (6 mm wide) filtration, respectively. The images are displayed using a red or blue color scale showing negative or positive pixels respectively.

The location of the HCC was defined on unenhanced CT according to the surgical records, and the slice with the largest lesion was selected by one of the research conductors (a radiologist with 22 years of experience with abdominal CT). Images were loaded onto a workstation for further texture analysis. Texture analysis was performed by a single observer (a radiologist with 12 years of experience with abdominal CT) who was blinded to the clinical outcome, using TexRAD (TexRAD Ltd., www.texrad.com part of Feedback Plc., Cambridge, UK), a proprietary research software algorithm developed to visualize and quantify the texture properties of tissues from medical imaging scans. The region of interest (ROI) was initially delineated around the tumor periphery section by an observer and refined by excluding areas of air using a thresholding procedure that removed any pixels with attenuation values below −50 Hounsfield unit from the analysis. The assessment of texture feature comprised an initial filtration step in which a Laplacian of Gaussian spatial band-pass filter was used to selectively extract features of different sizes and intensity variations, at three different frequency scales: fine (features approximately 2 mm in width), medium (features approximately 4 mm in width) and coarse (features approximately 6 mm in width) (Fig. 1). The heterogeneity within the ROI was assessed with and without filtration, and texture parameters were calculated as follows: the mean grey-level intensity (mean), variation/dispersion from the mean grey-level intensity (standard deviation, SD), the irregularity or complexity of the grey signal (entropy), the average intensity of the positive grey-level signal pixel values within the ROI (mean of the positive pixels, MPP), asymmetry of the distribution (skewness), and pointiness or peakedness of the distribution (kurtosis)^4,5^. These parameters derived from the histogram analysis. SD, kurtosis and skewness describe the shape of the histogram representing the gray-level variation, asymmetry and peak with in the ROI, respectively. Entropy is a measure of texture irregularity and defined by following equation where *l* is the pixel levels (between *l* = 1 to *k*) in ROI, and *p*(*l*) is the probability of the occurrence of that pixel level.

$${\rm{Entropy}}(e)=-\sum _{l=1}^{k}[p(l)]{\mathrm{log}}_{2}[p(l)]$$

Detailed information is described in^5^.

### Statistical analysis

The relationship between the CT texture features and 5-year overall survival (OS) or disease-free survival (DFS) was assessed by Kaplan-Meier analyses. Patients were dichotomized according to the best cut-off values, which were calculated by the log-rank test to classify the outcome of OS or DFS.

The multivariate Cox proportional hazard regression analysis using likelihood ratio test was performed to evaluate the independence of CT texture feature from clinical and pathological parameters, such as the Child-Pugh score, serum levels of AFP, tumor histological differentiation, pStage, or presence/absence of venous invasion, on OS or DFS. The correlations between CT texture features and clinical parameters were assessed: presence of HBs-Ag or HCV-Ab using paired-t-test; Child-Pugh classification, differentiation of HCC, or TNM stage using Spearman’s correlation; AFP using Pearson’s correlation. A *P*-value < 0.05 was considered to indicate statistical significance. The correlations of the CT texture features were assessed using the Spearman rank correlation coefficient. All statistical analyses were performed by a statistician using SPSS (version 18.0; IBM Corp, Armonk, NY, USA) statistical software.

## Results

Of the total number of patients, 12 were stage I, 60 stage II, 35 stage III, 12 stage IVA, and 3 stage IVB (Table 1). A total of 60 patients had a Child-Pugh score of 5, 46 a score of 6, 13 a score of 7, 2 a score of 8, and 1 a score of 9.Patients with the Child-Pugh score of 8–9 had inherited constitutional jaundice. AFP levels above 400 ng/ml were observed in 22 (18%) patients and venous invasion in 38 (31%) patients. A total of 77 (63.1%) patients had a single HCC, 24 (19.7%) patients had two HCCs, and 21 (17.2%) patients had three or more HCCs. The histopathologic differentiation of the tumors was well in 26 (21.3%) patients, moderate in 82 (67.2%) patients, poor in 10 (8.2%) patients and necrosis in 4 (3.3%) patients. Sixty-two (50.8%) patients died within 5 years, and the median OS was 50.6 months. A total of 98 (80.3%) patients suffered HCC relapse within 5 years, and the median DFS was 19.8 months.

**Figure 2:**
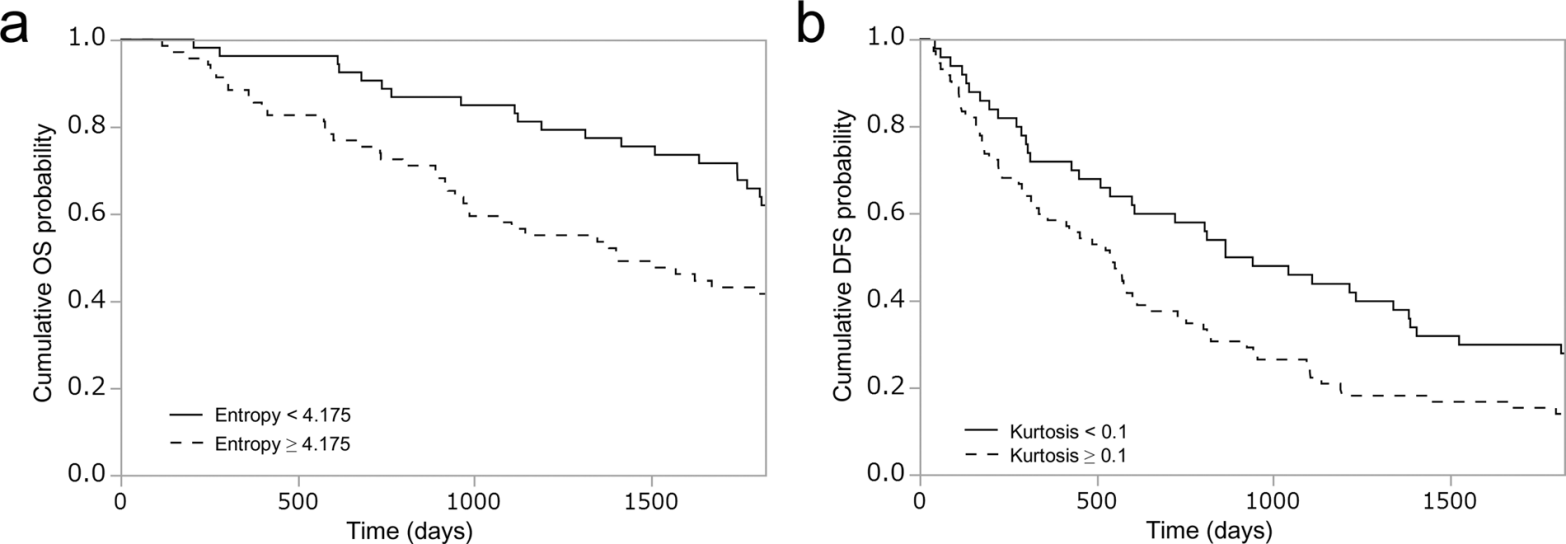
Kaplan-Meier curves for overall survival at fine filtration (**a**) and disease-free survival for kurtosis at coarse filtration (**b**).

The Kaplan-Meier curves for OS or DFS was significantly different between patient groups dichotomized by cut-off values for all CT texture parameters with filtration at at least single filter level (Table 2, Fig. 2). For SD and entropy, OS was significantly different without filtration or with filtration at each filter level (fine, medium and coarse). OS was different without filtration or with filtration (medium) for skewness. For mean, MPP and kurtosis, OS was not different without filtration between patient groups, but it was different with filtration (fine, fine and medium and coarse, and fine and medium, respectively). DFS was significantly different for skewness and kurtosis without filtration or with filtration (fine and medium, fine and coarse, respectively). DFS was different for mean, SD, entropy and MPP with filtration at single filter level (fine, coarse, coarse, and coarse, respectively).

**Table 2:** Results of Kaplan-Meier analysis for overall survival and disease-free survival.

Texture parameters and filter levels	Overall survival			Disease-free survival		
	Log-rank test		Likelihood ratio test	Log-rank test		Likelihood ratio test
	Threshold Value	*P*-value	*P*-value	Threshold Value	*P*-value	*P*-value
Mean						
no filtration	51.2	0.057	*0.035*	49.76	0.135	0.247
2 (fine)	−0.71	*0.007*	*0.002*	0.77	0.017	0.062
4 (medium)	−1.59	0.098	*0.04*	−11.68	0.111	0.054
6 (coarse)	−11.48	0.157	0.122	−22.35	0.084	0.053
SD						
no filtration	12.99	<*0.001*	<*0.001*	14.53	0.360	0.620
2 (fine)	18.05	*0.009*	*0.009*	24.88	0.250	0.131
4 (medium)	17.13	<*0.001*	<*0.001*	9.30	0.183	0.402
6 (coarse)	22.66	*0.003*	*0.003*	12.75	*0.002*	*0.009*
Entropy						
no filtration	4.01	<*0.001*	*0.001*	4.03	0.130	0.448
2 (fine)	4.26	*0.003*	*0.001*	4.27	0.270	0.381
4 (medium)	4.22	<*0.001*	*0.001*	4.33	0.117	0.243
6 (coarse)	4.49	<*0.001*	<*0.001*	4.53	*0.004*	*0.021*
MPP						
no filtration	51.2	0.057	*0.035*	49.76	0.135	0.247
2 (fine)	15.15	*0.003*	*0.001*	12.39	0.192	0.349
4 (medium)	16.21	*0.003*	<*0.001*	5.51	0.091	0.111
6 (coarse)	17.18	*0.006*	*0.001*	2.17	*0.036*	*0.019*
Skewness						
no filtration	−0.13	*0.011*	*0.019*	−0.13	*0.010*	*0.023*
2 (fine)	−0.11	0.119	0.111	−0.11	*0.049*	0.071
4 (medium)	−0.04	*0.005*	*0.01*	0.18	*0.027*	0.077
6 (coarse)	−1.47	0.097	0.237	0.69	0.071	0.093
Kurtosis						
no filtration	0.20	0.112	0.179	−0.13	*0.034*	*0.013*
2 (fine)	0.61	*0.027*	0.027	0.61	*0.015*	0.058
4 (medium)	−0.02	*0.047*	0.138	−0.21	0.177	0.475
6 (coarse)	1.38	0.162	0.287	1.37	*0.044*	*0.040*

SD, standard deviation; MPP, mean of the positive pixels.

Among the CT texture features and clinical and pathological parameters for OS, multivariate Cox proportional hazard regression analysis using likelihood test showed a significant effect of SD, entropy and MPP without filtration or with filtration at each filter level. For mean and skewness, a significant effect was found without filtration or filtration at single filter lever (fine, medium, respectively) (Table 2). SD, entropy and MPP at coarse filter, and skewness without filtration showed a significant effect for DFS. Thus, the possibility of CT texture feature increase the prognostic prediction of HCC by clinical and pathological information was shown. The correlation between CT texture features and clinical parameters (presence of HBs- Ag or HCV-Ab, Child-Pugh classification, differentiation of HCC, TNM stage or AFP) was not found (Table 3).

**Table 3:** Results of correlations between CT texture features and clinical parameters.

Texture parameters and filter levels	HBs-Ag	HCV-Ab,	Child-Pugh classification	HCC differentiation	TNM stage	AFP
	P-value of ﻿paired-t-test		Spearman’s correlation coefficient			Pearson correlation coefficient
Mean						
no filtration	0.41	0.23	−0.01	−0.14	−0.13	−0.06
2 (fine)	0.59	0.41	0.01	−0.04	0.05	0.05
4 (medium)	0.73	0.83	0.04	−0.04	0.03	0.04
6 (coarse)	0.76	0.71	0.07	−0.05	0.03	0.02
SD						
no filtration	0.50	0.73	−0.01	0.07	0.14	0.07
2 (fine)	0.73	0.36	0.05	0.05	0.14	0.03
4 (medium)	0.58	0.24	0.05	−0.06	−0.03	−0.02
6 (coarse)	0.41	0.12	0.03	−0.17	−0.10	−0.04
Entropy						
no filtration	0.47	0.33	0.00	0.06	0.11	0.11
2 (fine)	0.44	0.16	0.07	0.04	0.07	0.15
4 (medium)	0.74	0.11	0.07	−0.03	−0.03	0.07
6 (coarse)	0.43	0.08	0.06	−0.15	−0.09	0.05
MPP						
no filtration	0.45	0.27	−0.01	−0.14	−0.13	−0.07
2 (fine)	0.58	0.25	0.05	0.07	0.21	0.08
4 (medium)	0.58	0.58	0.13	−0.06	0.06	0.00
6 (coarse)	0.45	0.57	0.04	−0.08	−0.03	−0.03
Skewness						
no filtration	0.96	0.98	0.00	0.15	0.09	0.03
2 (fine)	0.94	0.16	0.02	−0.03	0.07	−0.04
4 (medium)	0.90	0.46	0.04	−0.04	0.03	−0.04
6 (coarse)	0.38	0.45	0.03	−0.01	−0.03	−0.10
Kurtosis						
no filtration	0.37	0.66	−0.12	−0.06	0.03	−0.02
2 (fine)	0.54	0.43	−0.07	−0.06	0.03	−0.03
4 (medium)	0.98	0.46	−0.04	−0.06	−0.13	0.05
6 (coarse)	0.94	0.18	−0.08	0.00	−0.12	0.05

**Figure 3:**
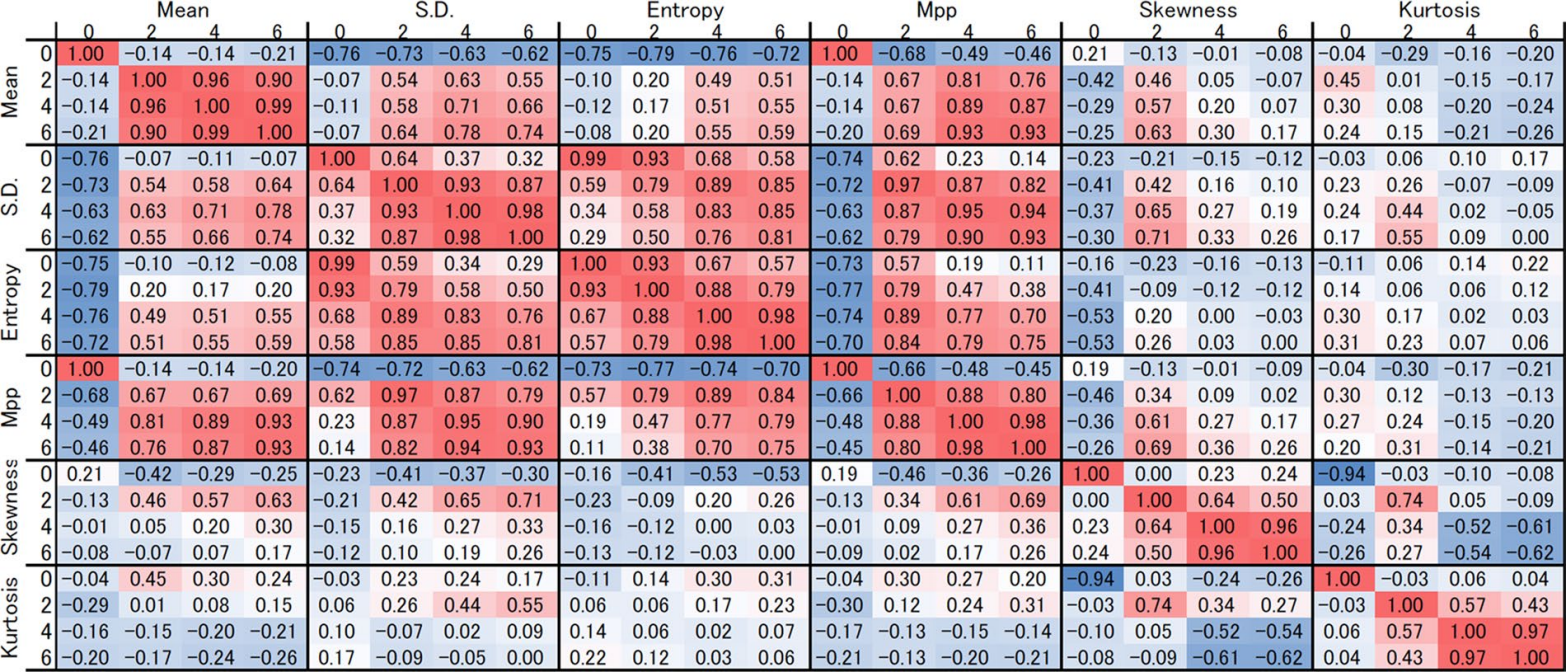
Correlation of each texture feature parameter.

Strong positive correlations were found between the SD and entropy at each filter level and without filtration, and the mean and MPP, mean and SD, SD and MPP, and entropy and MPP at each filtration (Fig. 3). The skewness or kurtosis was not strongly related to mean, SD, entropy or MPP.

## Discussion

In this study, we assessed the relationship between non-contrast-enhanced CT texture features and the prognosis of HCC. When patients were dichotomized using the cut-off calculated using the log-rank test, the Kaplan-Meier curves for OS or DFS was significantly different for CT texture feature parameters. Using multivariate Cox proportional hazard regression analysis, CT texture features showed a significant prognostic effect for OS or DFS similar on the clinical parameters.

The heterogeneity of a tumor is thought to be associated with variations in genomic subtype, gene expression, angiogenic factors, and the tumoral microenviroment^6^. This may explain the difficulties encountered in the validation of oncology biomarkers owing to sampling bias and help predict therapeutic resistance^7^. The assessment of heterogeneity has been applied to classifying pulmonary nodules^8–11^, assessing disease severity^12–14^, and determining the prognoses of colorectal carcinoma^15^, non-small cell lung cancer^16^, and esophageal carcinoma^17^.

We assessed the heterogeneity of HCC using texture analysis to determine the prognosis of HCC. Similar to previous reports^15–17^, we determined a relationship between the prognosis of HCC and the texture features of preoperative non-contrast-enhanced CT images. To reduce photon noise, which may mask any underlying biological heterogeneity, a Laplacian of Gaussian filter using an edge-detecting filter with Gaussian smoothing to remove high-frequency image photon noise was adopted. This filter was also adopted in the previous studies of tumor prognosis using CT^15–17^. The Kaplan-Meier curves for OS or DFS was significantly different between patient groups dichotomized by cut-off values for all CT texture parameters with filtration at at least single filter level. Without filtration, OS or DFS was not significantly different for some CT texture parameters. These results may show the CT texture analysis with filtration delineated the biological heterogeneity which related to the prognosis more in detail than without filtration. Texture analysis with filtration may be useful for developing preoperative strategies for HCC and personalized medicine.

SD, entropy, and MPP showed similar results; OS was different in the Kaplan-Meier curves at each filter level, and DFS was different at the coarse filter. The Spearman rank correlation coefficient revealed a high positive correlation among these CT texture parameters. On the other hand, skewness and kurtosis showed different trends from SD, entropy, and MPP. The skewness and kurtosis are a higher order of statics, whereas SD, entropy, and MPP are first or second order statics^4^. This may explain that skewness and kurtosis behave differently from other texture features. We applied three levels for filtration. Among them, coarse filter of SD, entropy and MPP showed significant difference among dichotomized patient groups for both of OS and DFS. Focusing on SD, entropy, and MPP, the coarse filter was useful to predict both of OS and DFS of HCC patients.

For SD, entropy, and MPP, DFS did not differ between dichotomized patients groups based on cut-off values at lower filters (fine and medium) nor without filtration. This may show that the effect of photon noise to obscure heterogeneity at lower filter level or without filtration is more prominent than at coarse filter. Meanwhile, OS was different at each filter level. The reason to explain this difference between DFS and OS is not clear; however, the higher ratio (80.3%) for HCC relapse than the ratio of deaths (50.8%) within 5 years could be one of the causes. The incidence of recurrence remains after hepatectomy, with a 5-year recurrence rate of approximately 80%^18–20^, and the data of this study are consistent with previous reports.

The clinical or pathological prognostic factors for HCC were the Child-Pugh score, AFP serum levels, pathological differentiation, pStage, or presence/absence of venous invasion^21–23^. The independence of texture features with respect to these parameters was evaluated by multivariate Cox proportional hazard regression analysis. We found independence of most CT texture parameters except kurtosis on OS, and a few CT texture parameters (SD, entropy and MPP with coarse filter, and skewness at without filtration) showed independence on DFS. These results were similar to results of Log-rank test. This indicates CT texture feature may increase the prognostic prediction of HCC by clinical and pathological information. For the prevention of HCC recurrence, several therapies, such as transarterial radioactive iodine, adoptive immunotherapy, use of retinoid, interferon and vitamin K2, are reported^24^. The information of the prognostic prediction of HCC is considered useful for the selection of patients undergoing these prophylactic therapies. Also, frequent examination for recurrence check may be performed on patients at high risk of recurrence.

We demonstrated that texture analysis using non-contrast-enhanced CT images show a relationship with HCC prognosis. CT assessment of preoperative HCC requires a contrast agent, except in cases with contraindications, such as an allergy to the iodinated contrast agent, asthma, or renal failure. Usually, the information gained from non-contrast-enhanced CT imaging for HCC is limited, and thus enhanced CT images are regarded as more useful for forming preoperative strategies for HCC. In this study, we showed the significance of non-contrast-enhanced CT images in the assessment of HCC prognosis. Texture analysis provided information on the prognosis of HCC, which has previously been unavailable with non-contrast-enhanced CT imaging. Recently, since a transitional stage has been initiated in radiological assessments such as radiomics^25,26^, the evaluation of HCC using texture analysis is expected to play a significant role in determining strategies for HCC. Enhanced CT provides information related to the vascularity of the lesion. Vascular heterogeneity may result in localized reduction in blood flow leading to areas of hypoxia, which may be associated with tumor progression^4^. Previous studies of the prognosis of tumor using CT texture features are performed using contras-enhanced CT^15–17^, therefore this study is considered to be unique in that non-contrast-enhanced CT is used. The vascular heterogeneity affected the assessment of texture features in the previous studies, whereas this study has not been affected by the information of vascularity. The comparison of texture analysis with and without vascular information is expected to discover new information of tumor. The role of enhanced CT in the prediction of HCC prognosis should be assessed in the near future.

There were some limitations to this study. First, only patients with preoperative HCCs were enrolled. Hepatectomy is the established first-line therapy for HCC^27–29^, however there are several other therapies for HCC such as radiofrequency ablation, TACE, and chemotherapies. The usefulness of texture analysis for the prediction of HCC prognosis should be assessed alongside these treatment options. Second, we excluded patients who underwent TACE before CT, because deposited iodized oil in the HCC may affect the CT texture analysis. Iodized oil deposits appear as high-density features on CT images and may affect the assessment of texture analysis. Preoperative TACE is not recommended routinely; however, TACE is used to reduce the tumor bulk in patients with HCC with borderline resectability, and increased tumor resectability appears to improve survival rates^30^. Thus, TACE is an important therapy for unresectable HCC^31,32^. The relationship between HCC heterogeneity and prognosis in patients undergoing TACE should be assessed, considering a large number of unresectable HCC cases. Third, the ROI was traced manually on a single slice containing the largest lesion. This manual process may induce bias in the tumor range; therefore, an automatic ROI drawing system is desirable. HCC is a three-dimensional structure; however, a three-dimensional analysis was not available on our workstation. The value of three-dimensional texture features for HCC should be assessed in the future. Fourth, multiple CT scanners were utilized in this study. The scanning parameters were controlled in a single institute, and the interscanner difference in the values of the radiomics features is known^33^. Filtration, which reduces the effect of photon noise in the assessment of texture features, may have a potential to affect the interscanner difference, and this should be assessed.

In conclusion, texture analysis of non-contrast-enhanced CT images using filtration showed a relationship with HCC prognosis. The possibility of CT texture feature increase the prognostic prediction of HCC by clinical and pathological information was shown. The texture features of HCC are expected to be novel information obtained from preoperative non-contrast-enhanced CT images.
